# Associations of cognitive impairment, functional decline and depression with the risk of all‐cause and cardiovascular mortality: a longitudinal study in the Chilean elderly population

**DOI:** 10.1002/alz.091530

**Published:** 2025-01-09

**Authors:** Paulina Orellana, Carlos Celis‐Morales, Claudia Duran‐Aniotz, Agustin Ibañez, Gabriela Nazar, Carolina Ochoa‐Rosales

**Affiliations:** ^1^ Latin American Institute for Brain Health (BrainLat), Universidad Adolfo Ibañez, Santiago Chile; ^2^ Center for Social and Cognitive Neuroscience (CSCN), School of Psychology, Universidad Adolfo Ibañez, Santiago Chile; ^3^ School of Cardiovascular and Metabolic Health, University of Glasgow, Glasgow United Kingdom; ^4^ Human Performance Lab, Education, Physical Activity and Health Research Unit, University Católica del Maule, Talca Chile; ^5^ Global Brain Health Institute (GBHI), Trinity College Dublin (TCD), Dublin Ireland; ^6^ Latin American Brain Health (BrainLat) Institute, Universidad Adolfo Ibáñez, Peñalolén, Santiago Chile; ^7^ Psychology Department, Social Sciences Faculty, Universidad de Concepción, Concepción Chile; ^8^ Latin American Brain Health Institute (BrainLat), Universidad Adolfo Ibañez, Santiago Chile

## Abstract

**Background:**

In Chile, the cases of cognitive impairment and dementia are on the rise and are expected to triple by 2050. Additionally, older adults face life changes that may contribute to depression onset. We studied the relationship between cognitive impairment (CI), functional decline (FD) and depression with the risk of all‐cause and cardiovascular event mortality in the Chilean population aged ≥60 years.

**Method:**

Prospective cohort study based on the Chilean National Health Survey 2009‐2010, a nationally representative prevalence study. Data on mortality status were available until 2020. We included n = 1,227 participants aged ≥60 years. A case of CI was defined as a score <13 in the abbreviated Mini‐Mental State Examination (score from 1‐19). FD in daily activities was defined as a score >6 in the Pfeffer Functional Activities Questionnaire test (score from 0‐33). Depression was defined as the presence of ≥5 depressive symptoms during the last year, assessed with the CIDI‐SF scale.

Survival probabilities were obtained through Kaplan‐Meier estimators. The associations of CI, FD, and depression (independent variables) with all‐cause and cardiovascular mortality (dependent variables) were investigated using multivariate Cox proportional hazard models. Results were expressed as Hazard Ratios and 95%CI (HR; 95%CI).

**Result:**

Participants were on average 71.7 years old and 60.3% were women. During the follow‐up, 431 individuals died from any cause and 107 from a cardiovascular event. Survival probabilities were lower among those with CI, FD and depression (Figure 1). CI was associated with higher risk of all‐cause and cardiovascular mortality by 60% (HR:1.60; 95%CI 1.25‐2.05) and 107% (2.07; 1.28‐3.34), respectively. FD and depression were associated with higher all‐cause mortality risk by 89% (1.89; 1.19‐3.01) and 69% (1.69; 1.25‐2.28), respectively. No significant associations were observed for FD and depression with cardiovascular mortality.

**Conclusion:**

This is the first study in the Chilean elderly population to link CI, FD or depression with a higher risk of premature mortality. Public health policies aimed to preserve mental and cognitive health in this age group are warranted to promote healthy aging.

